# Ablation in Pancreatic Cancer: Past, Present and Future

**DOI:** 10.3390/cancers13112511

**Published:** 2021-05-21

**Authors:** Govindarajan Narayanan, Dania Daye, Nicole M. Wilson, Raihan Noman, Ashwin M. Mahendra, Mehul H. Doshi

**Affiliations:** 1 Miami Cancer Institute, Baptist Health South Florida, Miami, FL 33176, USA; 2 Miami Cardiac and Vascular, Baptist Health South Florida, Miami, FL 33176, USA; 3 Herbert Wertheim College of Medicine, Florida International University, Miami, FL 33199, USA; nwils027@med.fiu.edu (N.M.W.); rnoma001@med.fiu.edu (R.N.); 4 Division of Vascular and Interventional Radiology, Department of Radiology, Massachusetts General Hospital, Harvard Medical School, Harvard University, Boston, MA 02114, USA; ddaye@mgh.harvard.edu; 5 Pratt School of Engineering, Duke University, Durham, NC 27708, USA; ashwin.mahendra@duke.edu; 6 Department of Radiology, Cleveland Clinic Florida, Weston, FL 33331, USA; doshim@ccf.org; 7 Charles E. Schmidt College of Medicine, Florida Atlantic University, Boca Raton, FL 33431, USA

**Keywords:** pancreatic cancer, interventional, oncology, ablation, IRE, microwave, cryoablation

## Abstract

**Simple Summary:**

Despite advancements in surgical oncology and chemoradiation therapies, pancreatic cancer still has one of the lowest 5-year survival rates in the United States. The silent progression of this disease often leads to it being identified after it has already reached an advanced and often unresectable stage. The field of interventional oncology has yielded ablation strategies with the potential to downstage and increase survival rates in patients suffering from locally advanced pancreatic cancer. This review examines the treatment strategies for locally advanced pancreatic cancer, discussing current results and future directions.

**Abstract:**

The insidious onset and aggressive nature of pancreatic cancer contributes to the poor treatment response and high mortality of this devastating disease. While surgery, chemotherapy and radiation have contributed to improvements in overall survival, roughly 90% of those afflicted by this disease will die within 5 years of diagnosis. The developed ablative locoregional treatment modalities have demonstrated promise in terms of overall survival and quality of life. In this review, we discuss some of the recent studies demonstrating the safety and efficacy of ablative treatments in patients with locally advanced pancreatic cancer.

## 1. Introduction

Pancreatic cancer is the fourth leading cause of cancer-related death in the United States, with a 5-year survival of approximately 9% [1]. Risk factors include smoking, obesity, and type 2 diabetes. Approximately 80–85% of the patients present with unresectable or metastatic disease. The American Cancer Society estimated that, in 2020, there will be 57,600 people diagnosed with pancreatic cancer and 47,050 related deaths. The high mortality of pancreatic cancer is primarily due to the insidious progression of this disease, resulting in a delayed diagnosis and often advanced disease at presentation. At the time of diagnosis, around 35% of patients have locally advanced disease (Stage III) and 50% of patients have metastatic disease (Stage IV). Furthermore, the aggressive tumor biology of pancreatic cancer contributes to early recurrence and metastasis in addition to resistance to chemotherapy and radiotherapy [2,3]. This review article is an overview of the current treatment options available in the management of pancreatic cancer, with a special focus on percutaneous ablation using irreversible electroporation and future directions.

## 2. Treatment Options for Locally Advanced Pancreatic Cancer

### 2.1. Surgery

Less than 20% of patients with pancreatic cancer are resectable at diagnosis. The most common surgery is the Whipple operation, where the pancreatic head, duodenum, gallbladder, and the antrum of the stomach are removed, with drainage of the distal pancreatic duct and biliary system accomplished through anastomosis to the jejunum. The overall mortality rate from a Whipple procedure is approximately 4–5%, with morbidity rates around 30–50% [4,5,6]. The most common postoperative complications include delayed gastric emptying occurring in 11–29% of patients, postoperative pancreatic fistula in 2–20%, postoperative abscess formation in 9–13%, bile leak in 2–8%, and post-pancreatectomy hemorrhage in 1–8% [4,7,8]. Variations include the pylorus sparing pancreaticoduodenectomy (PPPD) procedure, which is modified with the goal of improving nutritional deficiencies encountered in patients post-antrectomy [9]. A distal pancreatectomy is utilized in patients with tumors involving the body and the tail. A total pancreatectomy, which is a combination of both, is reserved for large tumors and has the highest associated mortality [9].

Surgical resection imparts improved survival to 23 months when a tumor-free margin of 1 mm (R0 resection) can be achieved. Survival is increased to 35 months when a greater than 1 mm tumor-free margin (R0 wide) can be achieved. The survival advantage of the resection is mostly lost when the tumor is detected within 1 mm of the resection margin. Of the approximately 15% of patients who undergo surgical resection, 5-year survival is 12% to 18% [1,2]. Patients undergoing curative resection for pancreatic cancer mostly develop recurrent tumor disease, with 69–75% of patients relapsing within 2 years and 80–90% relapsing within 5 years. The role of adjuvant and neoadjuvant treatments to improve surgical outcomes have been studied. The PRODIGE-24 ACCORD trial compared 6 months of adjuvant chemotherapy with modified FOLFIRINOX (FOLinic acid, Fluorouracil, IRINotecan and OXaliplatin) to gemcitabine monotherapy. The combination therapy group had increased disease-free survival of 21.6 months vs. 12.8 months and improved median overall survival of 54.4 months vs. 35 months [10].

The advantages of chemotherapy in the neoadjuvant setting include patient selection (those who progress with neoadjuvant therapy can avoid surgery), testing of chemosensitivity, higher rate of completion of systemic therapy, tumor control, and downstaging to R0 resection. The disadvantage is the toxicity of neoadjuvant treatment, which can limit the possibility of surgical resection. In the neoadjuvant setting, a meta-analysis by Petrelli et al. on FOLFIRINOX-based neoadjuvant therapy concluded that it resulted in a 39.4% R0 resection rate [11].

### 2.2. Chemotherapy

In patients with a good performance status, the standard of care for LAPC is the FOLFIRINOX or dose-attenuated modified FOLFIRINOX regimen [2,3,12]. A meta-analysis conducted by Suker et al. demonstrated that treatment-naïve patients with unresectable LAPC had a median overall survival (OS) of 24.2 months (95% CI, 21.7–26.8) and progression-free survival (PFS) of 15.0 months (95% CI, 13.8–16.2) when treated with FOLFIRINOX [3]. In this study, successful resection was possible following chemotherapy in approximately 25% of patients [3]. However, in the same meta-analysis, when comparing studies that evaluated adverse events, treatment with FOLFIRINOX resulted in a grade 3 or 4 adverse event rate of 60.4 events per 100 patients. For patients that cannot tolerate FOLFIRINOX treatment, second-line treatment options include combination therapy with gemcitabine and nab-paclitaxel. In those with a poor performance status, gemcitabine alone is the preferred chemotherapy [2,13]. While these chemotherapeutics confer a small increase in survival, the prognosis is still very poor in patients with unresectable LAPC.

### 2.3. Radiotherapy

The role of radiotherapy in LAPC has been controversial, with previous studies demonstrating equivalent or inferior results when used in conjunction with chemotherapy. In the FFCD/SFRO study, the authors investigated the use of adjuvant radiotherapy in LAPC by comparing chemoradiotherapy to chemotherapy alone in a randomized phase III clinical trial. Specifically, this study compared intensive induction chemoradiotherapy (60 Gy, infusional 5-FU and intermittent cisplatin) followed by maintenance gemcitabine to gemcitabine alone. Chemoradiotherapy was found to be more toxic and less effective than chemotherapy alone, with a median OS of 8.6 versus 13 months (*p* = 0.03), and toxicity (grades 3 and 4) of 36% versus 22% during the induction phase and 32% versus 18% during maintenance [14]. Another trial that compared chemoradiotherapy with chemotherapy was the LAP07 study. This was a two-stage randomization trial. In the first stage, patients were randomized to receive gemcitabine alone (*n* = 223) or gemcitabine with erlotinib (*n* = 219) for four months. Patients with progression-free disease were further randomized to receive two more months of the same chemotherapy (*n* = 136) or chemoradiotherapy (54 Gy plus capecitabine) (*n* = 133). No significant difference in the median OS was noted between chemotherapy (16.5 months) and chemoradiotherapy (15.2 months) (hazard ratio (HR), 1.03; 95% CI, 0.79–1.34; *p* = 0.83) [15]. Altogether, the FFCD/SFRO and LAP07 studies demonstrated that adjuvant radiotherapy has limited utility and possibly results in poorer outcomes in LAPC patients.

Advances in radiation include SBRT and MgRT. SBRT is delivered within five fractions and delivers a high dose to the tumor, with a steep falloff to much lower doses within a few millimeters outside of the tumor. The most recent publication on ablative radiation therapy and survival in inoperable pancreatic cancer patients and the role of SBRT included 119 consecutive patients treated with A-RT [16]. All patients with localized, unresectable, or medically inoperable pancreatic cancer with tumors of any size and less than 5 cm luminal abutment with the primary tumor were eligible. Ablative RT (98 Gy biologically effective dose) was delivered using standard equipment. Median OS from diagnosis and A-RT was 26.8 and 18.4 months, respectively. The study concluded that patients with inoperable LAPC treated with A-RT following multiagent induction therapy were associated with durable locoregional tumor control and favorable survival.

Magnetic resonance-guided radiation therapy (MRgRT) technology makes it possible to have continuous imaging of the tumor and nearby organs and automatically turns the beam on and off based on tumor positioning throughout respiration. MRgRT can safely deliver the ablative dose, which is at least twice as high as conventional SBRT. A recently published retrospective multicenter analysis using MRgRT showed that ablative doses of around the equivalent of 100 Gy using standard fraction sizes result in improved local control and OS when compared with the use of conventional lower doses [17].

### 2.4. Ablation

Despite advances in surgical techniques, chemotherapy, and radiation therapy, the incidence and mortality rate for pancreatic cancer have remained parallel to each other. This lack of substantial improvement points to aggressive cancer biology. The tumor microenvironment, particularly the interaction of the stromal elements with the cancer cells, has been postulated to confer resistance to treatment, early recurrence, and metastasis [18,19]. As a corollary to application in other solid organ tumors and to circumvent the resistance-conferring property of the tumor stroma, direct tumor treatment using ablative modalities has been attempted. Various ablative modalities (Table 1) used for pancreatic cancer include radiofrequency ablation, microwave ablation, cryoablation, high-intensity focused ultrasound, photodynamic therapy, and irreversible electroporation [20,21].

#### 2.4.1. Radiofrequency Ablation

Radiofrequency ablation (RFA) is a thermal ablative modality in which the application of alternative current in the radiofrequency range causes ionic agitation in the tumor, leading to frictional heat generation. Sufficient heat generation leads to tumor death by coagulative necrosis. RFA is one of the oldest ablative technologies and has the advantages of widespread availability and low cost. While there is extensive literature on the role of RFA in the treatment of primary and metastatic disease to the liver, the data on the use of RFA in primary pancreatic cancer are limited. Earlier studies with RFA were associated with high morbidity and mortality. Subsequent refinement in technique allowed further experience with RFA [20]. Ruarus et al. published a detailed review of ablative therapies in LAPC [21]. This review reported on six studies—each with more than ten patients—four of which were prospective and two that were retrospective. All six studies emanated from the same institution, with a percutaneous approach used only in one study. In the remaining five studies, ablation was performed during laparotomy with a likely overlapping patient population. Overall survival was reported in four of these studies, with a range of 19.0 to 25.6 months. Morbidity and 30-day mortality were in the range of 0–28% and 0–3%, respectively. Serious adverse events included pancreatic fistula, acute pancreatitis, portal vein thrombosis, duodenal injury, biliary injury, gastric ulcer or fistula, hemoperitoneum, and liver failure. Causes of mortality were hepatic failure, sepsis following a duodenal perforation, severe acute pancreatitis, and duodenal hemorrhage. These studies, albeit limited, suggest that RFA is an option for treating unresectable LAPC.

#### 2.4.2. Microwave Ablation

Microwave ablation (MWA) uses alternating electromagnetic pulses in the microwave range, causing dielectric heating of the water molecules. As in RFA, coagulative necrosis is the mechanism of tumor destruction. Compared to RFA, microwave ablation offers advantages of active heating, larger ablations in shorter time, and does not require the placement of a grounding pad. Limited data are available on the application of MWA in pancreatic cancer. Lygidakis et al. published a retrospective study of 15 patients undergoing MWA during laparotomy. They reported partial necrosis in all patients, with no major morbidity or mortality. Minor complications were reported in 6/15 patients and included mild pancreatitis, asymptomatic hyperamylasemia, pancreatic ascites, and minor bleeding. Survival data were not reported [22]. In another retrospective study of ten patients (five percutaneous, five laparotomy), Carrafiello et al. reported 100% efficacy, with 9-month and 1-year local tumor progression rates of 37.5% (3/8) and 62.5% (5/8), respectively. In this study, they had two patients with minor complications and two major adverse events (pancreatitis and pseudoaneurysm of the gastroduodenal artery) [23].

Although a direct comparison of RFA and MWA has not yet been performed, results of single-arm retrospective studies hint towards more favorable outcomes with MWA in terms of safety and feasibility. One such study by Vogl et al. conducted retrospective reviews of computed tomography (CT)-guided percutaneous MWA using a 2.45 GHz device in 20 patients, and 22 tumors/sessions were published [24]. Seventeen tumors were in the pancreatic head and five in the pancreatic tail, with a mean tumor diameter of 30 ± 6 mm. In this study, there was a technical success rate of 100% with no major adverse events, with only two patients reporting severe local pain post-ablation. At a short 3-month imaging follow-up, progression was seen in only 1 out of 10 tumors with available follow-up imaging. While survival data are not currently available to evaluate the efficacy of MWA, this study suggests that MWA is a safer option for locoregional therapy of LAPC.

#### 2.4.3. Cryoablation

In cryoablation, argon gas is passed through a needle core utilizing the Joule–Thomson effect; rapid freeze and thaw cycles lead to cellular destruction mediated by cell membrane disruption, vascular damage, and ischemia [21]. Cryoablation offers the advantage of multiprobe placement, visualization of the ice-ball or ablation zone, and being MRI compatible. In LAPC, outcomes of cryoablation plus bypass surgery were compared with those of bypass surgery alone in two separate studies [25,26]. Despite tumor shrinkage in the cryoablation plus bypass surgery group, OS was not statistically different (350 days versus 257 days, *p* = 0.124: 5 months versus 4 months, *p* > 0.05). Patients receiving cryoablation experienced higher incidence of post-operative delayed gastric emptying. Postoperative complications included pancreatic or bile leak, gastrointestinal bleeding or obstruction, delayed gastric emptying, infection, or intra-abdominal bleeding [25,26].

The utility of cryoablation for treating pancreatic cancer via the percutaneous approach has been assessed by Niu et al. in a retrospective review of 32 patients undergoing 49 ablations [27]. Tumor size ranged from 2 to 11 cm, with a mean of 5.2 cm ± 8 cm. Disease stage distribution included stage II (*n* = 3), stage III (*n* = 11), and stage IV (*n* = 18), with no patient deaths or major complications in the study. This study demonstrated a significant reduction in pain and analgesics requirement post-cryoablation, along with an improvement in performance status. Additionally, mean and median survival was 15.9- and 12.6-months post-treatment, with the 6-, 12-, and 24-month survival rates at 82.8%, 54.7%, and 27.3%, respectively. The absence of complications and reduction in cancer-related pain in this study suggest that cryoablation is a palliative option for unresectable LAPC. Limitations include the limited experience with the technology and the dearth of high-quality data.

#### 2.4.4. Photodynamic Therapy

In photodynamic therapy (PDT) visible or near-infrared light is used to activate a light-sensitive drug or photosensitizer, (PS) which, in the presence of ground state oxygen, creates reactive oxygen species and radicals that can induce tissue death [28,29]. Light is delivered via small optic fibers placed percutaneously under image guidance. The first phase I trial of PDT in locally advanced PDAC was conducted in 2002 by Bown et al. [30]. Tumor necrosis was achieved in all 16 patients included in the study. Median survival after PDT was 9.5 mo (range 4–30 mo), and 44% (7/16) were alive one year after PDT. There was tumor regrowth from the edges of treated areas. One of the drawbacks of the early PDT treatments was that patients had to spend several days in subdued lighting following the treatment to prevent complications from skin necrosis.

Verteporfin (Visudyne™, Valeant Pharmaceuticals, Bridgewater, NJ, USA) is a vascular-targeted PS and can be administered just one hour before light treatment, and patients are light-sensitive for only 24 h after treatment. Huggett et al. describe the results of a phase I/II trial of verteporfin PDT on locally advanced pancreatic cancer [31]. Fifteen patients with locally advanced cancers in the head of the pancreas and who could not undergo surgical resection were given 0.4 mg/kg verteporfin. Necrotic volumes were determined for all patients, which increased with increasing light dose. The median survival after I-PDT was 8.8 months and was comparable to patients undergoing conventional treatment. The median survival from diagnosis was 15.5 months.

#### 2.4.5. Irreversible Electroporation

**Figure 1:**
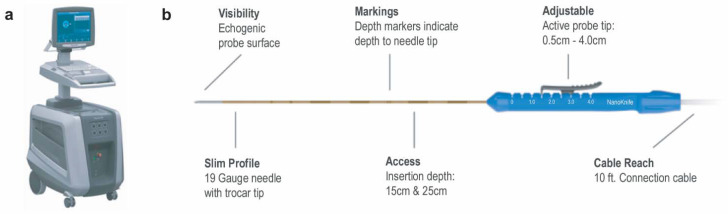
(**a**) The NanoKnife System. (**b**) NanoKnife IRE probe, 19-gauge needle with trocar tip with adjustable active probe tip ranging from 0.5 to 4.0 cm.

Irreversible electroporation (IRE) uses a high-voltage, low-energy DC current as the energy source, causing cellular injury by the creation of irreversible nanoscale defects in the phospholipid bilayer of the cellular membrane (Figure 1), resulting in cellular apoptosis [32,33]. IRE is primarily nonthermal and can achieve destruction of tumors with sharp transition zones. Additionally, it spares vascular, ductal, and connective tissue structures while not being vulnerable to the heat-sink effect and can be used close to blood vessels [34].

IRE in the pancreas was initially studied in a swine model by Charpentier et al. and was concluded to be a safe method for pancreatic tissue ablation [35]. The alterations to the tumor microstructure in pancreatic cancer following irreversible electroporation ablation were studied in a mouse model [36]. The purpose of this study was to elucidate morphological alterations following 30 min of IRE ablation in a mouse model of pancreatic cancer. Early-passage PANC-1 cells were harvested and were implanted into both flanks of 14 mice. A total of 3–4 weeks were allowed for tumor growth to a size of approximately 8 mm (longest diameter for each tumor measured using calipers) prior to IRE procedures. These mice were randomly divided into two groups: for the first group (*n* = 8), left-flank tumors went untreated while right-flank tumors were treated with the IRE ablation protocol; for the second group (*n* = 6), both left- and right-flank tumors were treated with IRE ablation. Immunohistochemistry markers were compared with diffusion-weighted MRI (DWI) apparent diffusion coefficient measurements before and after IRE ablation. Immunohistochemistry apoptosis index measurements were significantly higher in IRE-treated tumors than in controls. Rapid tissue alterations after 30 min of IRE ablation procedure, consisting of structural and morphological alterations along with significantly elevated apoptosis markers, were observed and correlated with apparent diffusion coefficient measurements. This imaging assay demonstrated the potential to serve as an in vivo biomarker for the noninvasive detection of tumor response in the pancreas following IRE ablation.

The initial human data on the use of IRE in pancreatic cancer were from the surgical literature using an open approach [37]. This was followed by the percutaneous technique using CT guidance. The first published percutaneous data in humans were from a retrospective cohort of 14 patients who had 15 ablations [38]. Three patients had metastatic disease and eleven had LAPC. All patients had received chemotherapy previously, and eleven had received radiation. The median tumor size was 3.3 cm (range, 2.5–7 cm). Two patients were downstaged to surgery 4 and 5 months after IRE, respectively. Both had margin-negative resections, and one had a complete pathologic response. This study established the feasibility of treating pancreatic cancer percutaneously and was followed by another retrospective review of 50 patients with LAPC treated with percutaneous IRE [39]. The primary objective was safety, and the secondary objective was overall survival. Median OS was 27 months (95% CI, 22.7–32.5 months) from the time of diagnosis and 14.2 months (95% CI, 9.7–16.2 months) from the time of IRE. On multivariate analysis, tumors < 3 cm had an OS significantly longer than those >3 cm, 33.8 vs. 22.7 months from the time of diagnosis, and 16.2 vs. 9.9 months from IRE.

Edward Leen et al. published a retrospective review of seventy-five patients with unresectable pancreatic carcinoma who underwent percutaneous IRE after chemotherapy between 2011 and 2016 [40]. Median OS and PFS post-IRE for LAPC were 27 and 15 months, respectively. Four patients with LAPC downstaged post-IRE ablation to surgery, with R0 resections in three cases.

The open and percutaneous approach of IRE in the pancreas were studied in a retrospective review of eight patients, where four were treated with an open approach and four percutaneously with ultrasound guidance [41]. There were no patient deaths 90 days post-IRE. There were five minor complications in three patients and four major complications in three patients. The incidence of complications did not differ between approaches. The median OS was 17.5 months from IRE and 24 months from diagnosis.

**Figure 2:**
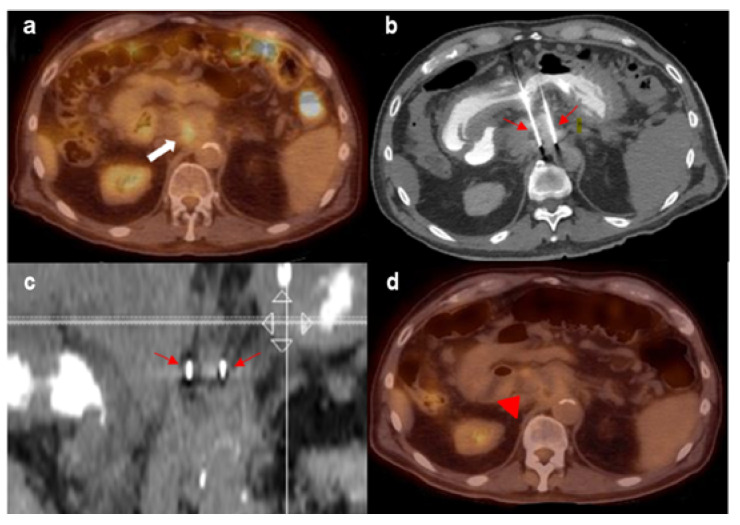
(**a**) FDG-avid recurrent pancreatic mass (white thick arrow). (**b**,**c**) Two IRE probes in place bracketing the mass (thin red arrows). (**d**) No residual FDG avidity post-treatment (red arrow).

Figure 2 demonstrates a case example using IRE to treat unresectable LAPC. A 72-year-old man, initially diagnosed with pancreatic adenocarcinoma in 2014, underwent chemotherapy and IRE in 2014, and had external radiation to the neck of the pancreas. Follow-up PET/CT 5 years later demonstrated FDG uptake within an ill-defined soft tissue density inseparable from the pancreatic head at the level of the celiac trunk, concerning for tumor recurrence. Patient also had elevated CA-19–9 levels. Repeat IRE treatment was performed with two probes, with no residual FDG uptake in the post-treatment PET.

#### 2.4.6. Prospective IRE Trials

The promising results from retrospective IRE studies opened the door for prospective trials, the most notable being the PANFIRE trials (Percutaneous Irreversible Electroporation in Locally Advanced and Recurrent Pancreatic Cancer). The PANFIRE I/II study, reported a 12-month median time to local progression after percutaneous IRE (95% CI: 8–16 months) [42]. The median OS was 11 months from IRE (95% CI: 9–13 months) and 17 months from diagnosis (95% CI 10–24 months) [42]. This was followed by PANFIRE II, a multicenter, prospective, single-armed trial consisting of 50 patients diagnosed with locally recurrent or advanced pancreatic cancer [43]. Patients in this trial demonstrated a median OS of 10 months from IRE (95% CI: 8–11 months) and median OS of 17 months from diagnosis (95% CI: 15–19 months). In addition to the possible survival benefit of IRE, the patients who received FOLFIRINOX before IRE did not demonstrate a survival benefit compared with those who received gemcitabine or no chemotherapy before IRE (HR = 1.1; *p* = 0.70). These results suggest that IRE was the key determinant for improved survival.

Additional work by He et al. compared the survival benefit in post-induction chemotherapy patients treated with IRE vs. those treated with radiotherapy [44]. In this study, thirty-six pairs of patients with LAPC were selected through propensity score matching (PSM) analysis to compare the efficacy of the two treatments. After PSM analysis, a survival benefit was identified in the IRE-treated group when compared to the radiotherapy-treated group, with an improvement in both PFS and OS (2-year OS was 53.5% vs. 20.7%, *p* = 0.011; 2-year PFS rates were 28.4% vs. 5.6%, *p* = 0.004). Multivariate Cox regression analysis indicated that IRE after induction chemotherapy was identified as a single favorable factor for both OS and PFS in both the whole and matched cohort.

A more recent prospective study enrolled 54 patients (30 men; median age 61.0 years; range 41–73 years) undergoing IRE with or without chemotherapy for pancreatic cancer between July 2015 and August 2016 [45]. Thirty-one patients received IRE + chemotherapy and 23 had IRE alone. Major IRE-related complications were observed in four patients (7.4%). Among those with stage III disease, after a median follow-up of 18.8 months (range 9.6–28.7 months), the median OS from diagnosis was 16.2 and 20.3 months in the IRE and IRE + Chemo groups, respectively. Among those with stage IV disease, after a median follow-up of 13.3 months (range 3.7–23.1 months), the median OS from diagnosis was 11.6 and 13.56 months in the IRE and IRE + Chemo groups, respectively. The OS was significantly poorer in the IRE group than in the IRE + Chemo group (log-rank test, *p* = 0.0398) [45].

CROSSFIRE (ClinicalTrial.gov, NCT02791503), a randomized, controlled, phase III trial, is currently recruiting patients in Europe, comparing the outcome of FOLFIRINOX plus IRE with FOLFIRINOX plus stereotactic ablative radiotherapy on OS for patients with LAPC.

In the United States, the FDA provided the breakthrough designation status for IRE in pancreatic cancer in 2018. This was followed by the investigational device exemption approval, which paved the way for the first prospective multicenter trial from the US to study IRE in a randomized, controlled fashion. This multicenter trial will involve 264 patients receiving IRE and 264 patients receiving standard of care treatment. The primary endpoints will be OS and safety. Other endpoints will include PFS and pain scores. Following induction chemotherapy with FOLFIRINOX, patients with inoperable biopsy proven stage III pancreatic carcinoma will be randomized to IRE plus institutional standard of care treatment and follow-up vs. institutional standard of care treatment and follow-up. The IRE treatment can be offered by either a percutaneous or open surgical approach. The study is currently enrolling (ClinicalTrial.gov, NCT03899636).

## 3. Post-Ablative Imaging in Pancreatic Cancer

With the advent and adoption of current locoregional treatments and future immune-modulating agents, imaging response post-locoregional therapy in the pancreatic cancer has not kept up with improvements in the treatment. The Response Evaluation Criteria in Solid Tumors (RECIST) and RECIST 1.1 criteria have been used extensively since their introduction, and concerns about using change in tumor size as the only criterion have not been fully addressed, even in RECIST 1.1.

Pancreatic cancer is a desmoplastic neoplasm, hypo-vascularized and rich in fibrous tissue, making it difficult to distinguish between residual/recurrent tumor and simple fibrotic tissue. Katz et al. showed that the response of borderline resectable pancreatic cancer to neoadjuvant therapy is not reflected by radiographic indicators and RECIST response was not an effective treatment endpoint for patients with borderline resectable pancreatic cancer [46].

Vroomen et al. studied the MR and CT imaging characteristics and ablation zone volumetry of LAPC treated with IRE and concluded that the most remarkable signal alterations after pancreatic IRE were shown by DWI and contrast-enhanced MRI. Integrated PET/MRI has been shown to provide an early response assessment in patients with advanced pancreatic ductal adenocarcinoma (PDAC) [47]. A combination of MR and PET scan along with markers will help to define the criteria to assess response to locoregional treatment to the pancreas.

## 4. The Future of IRE

### 4.1. Immunomodulatory Effects of IRE

The stroma in pancreatic cancer prevents drug concentration and may be a reason for the relatively poor response to chemotherapy in pancreatic cancer [48]. Immunotherapy and immune checkpoint blockade have shown promise in different cancers but have had limited efficacy against pancreatic cancer, and one of the proposed reasons is the immunosuppressive stromal tissue. The stellate cells interact with cancer cells to modulate cell proliferation and extracellular matrix production, evade immune surveillance, and promote angiogenesis.

In a murine model study, IRE modulated the stroma, induced immunogenic cell death, activated dendritic cells, and alleviated stroma-induced immunosuppression. IRE reversed the resistance immune checkpoint blockade in pancreatic cancer and the study concluded that IRE is a promising approach to potentiate the efficacy of immune checkpoint blockade in pancreatic cancer [48].

Immune response following cryoablation and IRE was studied in a mouse model of pancreatic cancer, and IRE was found to evoke a more robust infiltration of macrophages and T cells than cryoablation within 24 h [49]. These recent results from animal studies offer a glimpse into the future potential of IRE in mediating or enhancing the immune response to pancreatic cancer. These preliminary animal studies provide a signal of the future potential role of IRE and immunotherapies in pancreatic cancer.

### 4.2. High-Frequency IRE—An Emerging Technique

High-frequency irreversible electroporation (HFIRE) is an emerging technique that uses bipolar square waves of 1–5 μs in rapid bursts, eliminating the need for intraoperative paralytics and cardiac synchronization. This technique addresses the current limitations associated with IRE by reducing the risks for cardiac asynchrony and muscle tetany. HFIRE can also be performed with single-needle, dual-electrode devices, which eliminate the need for a high level of technical skill typical of IRE to ensure accurate placement and alignment of multiple electrodes for optimal treatment.

A preclinical feasibility study of a next-generation, single-needle, high-frequency irreversible electroporation (SN-HFIRE) for pancreatic ablation has been performed using an in vivo swine model. Six swine pancreases were treated with open SN-HFIRE without intraprocedural paralytic medications or cardiac synchronization using varying voltage waveform settings (on-off-on, 1-5-1, 2-5-2, or 5-5-5 μs, with a total energized time of 100 μs) [50]. This is a departure from the current IRE device, which requires paralytics and cardiac synchronization. Key measured outcomes included intraprocedural muscle twitch and cardiac activity, and ablation zone size and volume 6 h post-procedure. All ablations were technically successful, with no change in muscle twitch or cardiac activity at any pulse setting during the 5 min ablations and larger ablation areas (85 vs. 41/44 mm^2^) and volumes (3344 vs. 1162 and 1339 mm^3^) using the 5-5-5 μs pulse setting. This feasibility study supports the ability of SN-HFIRE to create rapid ablation zones in pancreatic tissue without intraprocedural paralytic medications or cardiac synchronization.

Similarly, another recent preclinical study evaluated the safety and feasibility of single-needle HFIRE for the treatment of hepatocellular carcinoma (HCC) in a canine model [51]. Three canine patients with resectable HCC were treated with HFIRE (on-off-on, 2-5-2 μS with a total energized time of 100 μS) without cardiac synchronization or intraoperative paralytics. The authors evaluated the canine patients for adverse events and measured the ablation volumes on post-treatment CT and the treatment-specific lethal thresholds for malignant and healthy liver tissue; they also assessed the HFIRE-induced local immune response. Their results showed that HFIRE can be performed safely and effectively and resulted in predictable ablation volume (average volume of 3.89 cm^3^ ± 0.74). The lethal threshold was 710 V/cm ± 28.2. There were no adverse events reported and no cardiac asynchrony or muscle tetany. Immunohistochemical analysis also demonstrated a reactive proinflammatory margin surrounding the ablation zone, composed of collagen and CD3+/CD4−/CD8− lymphocytes. This study further supported the safety and feasibility of HFIRE and provided preliminary evidence that HFIRE is associated with a predictable ablation volume and lymphocytic tumor infiltration.

Both studies provide promising results supporting the feasibility of this technique, and they pave the way for broader applications of IRE, especially in patients who currently cannot be treated with this technique due to cardiac contraindications. Nonetheless, larger studies are still needed to validate these findings.

## 5. Conclusions

Pancreatic cancer is a complex disease and high-volume centers address this in a multidisciplinary manner. The silent nature of pancreatic cancer and presentation leaves a very small percentage of patients qualifying for surgery. Decades of conventional treatment of pancreatic cancer, initially with chemotherapy and chemoradiotherapy, have not made a significant impact on the overall survival of these patients. The synergistic capabilities of local ablative therapies with imaging guidance are an attractive, minimally invasive option which can be used in combination with traditional methods of treating pancreatic cancer. One of the main limitations of all these innovative treatment options is the paucity of level-one data. Among the several local ablative modalities reviewed, IRE has been shown to be safe and effective, demonstrating a signal towards improved survival in several studies. The prospective IRE trials that have been completed and are currently underway overcome the limitation of the lack of randomized controlled trial data. The results of the trials will help to answer some crucial questions regarding the role of this technology in the treatment algorithm of pancreatic cancer.

## Figures and Tables

**Table 1:** Comparison of ablative modalities in pancreatic cancer.

Procedure	Mechanism of Action	Advantages	Limitations
Radiofrequency ablation (RFA)	Utilizes alternating electrical current to create a thermal ablation zone in the tissue.	Inexpensive compared to other ablation modalities. Widespread availability.	Serious adverse events include pancreatic fistula, acute pancreatitis, portal vein thrombosis, duodenal injury, biliary injury, gastric ulcer or fistula, hemoperitoneum, and liver failure.
Microwave ablation (MWA)	Creates a zone of ablation by generating frictional heat through agitation of water molecules.	Relatively larger ablation zones and faster ablation times compared to RFA. Does not require grounding pads.	Possible adverse events include mild pancreatitis, asymptomatic hyperamylasemia, pancreatic ascites, and minor bleeding.
Cryoablation	Utilizes changes in argon gas pressure to generate freeze–thaw cycles (i.e., the Joule–Thomson effect) in the tissue. The probe tip can reach temperatures as low as −185 °C, inducing cell death in the surrounding tissue.	Good visualization of ice ball, and less postoperative pain and damage to nearby critical structures when compared to RFA and MWA.	Possible postoperative complications include pancreatic or bile leak, gastrointestinal bleeding or obstruction, delayed gastric emptying, infection, or intra-abdominal bleeding.
Irreversible electroporation (IRE)	Nonthermal process uses DC surrent to create permanent pores in cell membrane, increasing the permeability and inducing cell death.	Nonthermal and is not limited by the heat-sink effect like the thermal ablation modalities. Preferred method when working near vascular and other critical structures.	Risk for generating cardiac arrhythmias. Need for general anesthesia with cardiac monitoring and muscle relaxation. Probe placement has to be parallel.

## References

[1] Siegel R.L., Miller K.D., Jemal A. (2020). Cancer statistics, 2020. CA Cancer J. Clin..

[2] Kamisawa T., Wood L.D., Itoi T., Takaori K. (2016). Pancreatic cancer. Lancet.

[3] Suker M., Beumer B.R., Sadot E., Marthey L., Faris J.E., Mellon E.A., El-Rayes B.F., Wang-Gillam A., Lacy J., Hosein P.J. (2016). FOLFIRINOX for locally advanced pancreatic cancer: A systematic review and patient-level meta-analysis. Lancet Oncol..

[4] Gervais D.A., Fernandez-del Castillo C., O’Neill M.J., Hahn P.F., Mueller P.R. (2001). Complications after Pancreatoduodenectomy: Imaging and Imaging-guided Interventional Procedures. Radiographics.

[5] Sohn T.A., Yeo C.J., Cameron J.L., Geschwind J.F., Mitchell S.E., Venbrux A.C., Lillemoe K.D. (2003). Pancreaticoduodenectomy: Role of interventional radiologists in managing patients and complications. J. Gastrointest. Surg..

[6] Malgras B., Duron S., Gaujoux S., Dokmak S., Aussilhou B., Rebours V., Palazzo M., Belghiti J., Sauvanet A. (2016). Early biliary complications following pancreaticoduodenectomy: Prevalence and risk factors. HPB.

[7] Bassi D.C., Butturini G. (2005). Postoperative pancreatic fistula: An international study group (ISGPF) definition. Surgery.

[8] Wente M.N., Veit J.A., Bassi C., Dervenis C., Fingerhut A., Gouma D.J., Izbicki J.R., Neoptolemos J.P., Padbury R.T., Sarr M.G. (2007). Postpancreatectomy hemorrhage (PPH)–An International Study Group of Pancreatic Surgery (ISGPS) definition. Surgery.

[9] Bachmann J., Michalski C.W., Martignoni M.E., Büchler M.W., Friess H. (2006). Pancreatic resection for pancreatic cancer. HPB.

[10] Conroy T., Hammel P., Hebbar M., Ben Abdelghani M., Wei A.C., Raoul J.L., Choné L., Francois E., Artru P., Biagi J.J. (2018). FOLFIRINOX or Gemcitabine as Adjuvant Therapy for Pancreatic Cancer. N. Engl. J. Med..

[11] Petrelli F., Coinu A., Borgonovo K., Cabiddu M., Ghilardi M., Lonati V., Aitini E., Barni S., Gruppo Italiano per lo Studio dei Carcinomi dell’Apparato Digerente (GISCAD) (2015). FOLFIRINOX-based neoadjuvant therapy in borderline resectable or unresectable pancreatic cancer: A meta-analytical review of published studies. Pancreas.

[12] Thibodeau S., Voutsadakis I.A. (2018). FOLFIRINOX Chemotherapy in Metastatic Pancreatic Cancer: A Systematic Review and Meta-Analysis of Retrospective and Phase II Studies. J. Clin. Med..

[13] Von Hoff D.D., Ervin T., Arena F.P., Chiorean E.G., Infante J., Moore M., Seay T., Tjulandin S.A., Ma W.W., Saleh M.N. (2013). Increased survival in pancreatic cancer with nab-paclitaxel plus gemcitabine. N. Engl. J. Med..

[14] Chauffert B., Mornex F., Bonnetain F., Rougier P., Mariette C., Bouché O., Bosset J.F., Aparicio T., Mineur L., Azzedine A. (2008). Phase III trial comparing intensive induction chemoradiotherapy (60 Gy, infusional 5-FU and intermittent cisplatin) followed by maintenance gemcitabine with gemcitabine alone for locally advanced unresectable pancreatic cancer. Definitive results of the 2000-01 FFCD/SFRO study. Ann. Oncol..

[15] Hammel P., Huguet F., van Laethem J.L., Goldstein D., Glimelius B., Artru P., Borbath I., Bouché O., Shannon J., André T. (2016). Effect of Chemoradiotherapy vs Chemotherapy on Survival in Patients with Locally Advanced Pancreatic Cancer Controlled After 4 Months of Gemcitabine with or without Erlotinib: The LAP07 Randomized Clinical Trial. JAMA.

[16] Reyngold M., O’Reilly E.M., Varghese A.M., Fiasconaro M., Zinovoy M., Romesser P.B., Wu A., Hajj C., Cuaron J.J., Tuli R. (2021). Association of Ablative Radiation Therapy with Survival Among Patients with Inoperable Pancreatic Cancer. JAMA Oncol..

[17] Rudra S., Jiang N., Rosenberg S.A., Olsen J.R., Roach M.C., Wan L., Portelance L., Mellon E.A., Bruynzeel A., Lagerwaard F. (2019). Using adaptive magnetic resonance image-guided radiation therapy for treatment of inoperable pancreatic cancer. Cancer Med..

[18] Haqq J., Howells L.M., Garcea G., Metcalfe M.S., Steward W.P., Dennison A.R. (2014). Pancreatic stellate cells and pancreas cancer: Current perspectives and future strategies. Eur. J. Cancer.

[19] McMillin D.W., Negri J.M., Mitsiades C.S. (2013). The role of tumour-stromal interactions in modifying drug response: Challenges and opportunities. Nat. Rev. Drug Discov..

[20] Keane M.G., Bramis K., Pereira S.P., Fusai G.K. (2014). Systematic review of novel ablative methods in locally advanced pancreatic cancer. World J. Gastroenterol..

[21] Ruarus A., Vroomen L., Puijk R., Scheffer H., Meijerink M. (2018). Locally Advanced Pancreatic Cancer: A Review of Local Ablative Therapies. Cancers.

[22] Lygidakis N.J., Sharma S.K., Papastratis P., Zivanovic V., Kefalourous H., Koshariya M., Lintzeris I., Porfiris T., Koutsiouroumba D. (2007). Microwave ablation in locally advanced pancreatic carcinoma--A new look. Hepatogastroenterology.

[23] Carrafiello G., Ierardi A.M., Fontana F., Petrillo M., Floridi C., Lucchina N., Cuffari S., Dionigi G., Rotondo A., Fugazzola C. (2013). Microwave ablation of pancreatic head cancer: Safety and efficacy. J. Vasc. Interv. Radiol..

[24] Vogl T.J., Panahi B., Albrecht M.H., Naguib N.N.N., Nour-Eldin N.A., Gruber-Rouh T., Thompson Z.M., Basten L.M. (2018). Microwave ablation of pancreatic tumors. Minim. Invasive Ther. Allied Technol..

[25] Li J., Chen X., Yang H., Wang X., Yuan D., Zeng Y., Wen T., Yan L., Li B. (2011). Tumour cryoablation combined with palliative bypass surgery in the treatment of unresectable pancreatic cancer: A retrospective study of 142 patients. Postgrad. Med. J..

[26] Song Z.G., Hao J.H., Gao S., Gao C.T., Tang Y., Liu J.C. (2014). The outcome of cryoablation in treating advanced pancreatic cancer: A comparison with palliative bypass surgery alone. J. Dig. Dis..

[27] Niu L., He L., Zhou L., Mu F., Wu B., Li H., Yang Z., Zuo J., Xu K. (2012). Percutaneous ultrasonography and computed tomography guided pancreatic cryoablation: Feasibility and safety assessment. Cryobiology.

[28] Henderson B.W., Dougherty T.J. (1992). How does photodynamic therapy work?. Photochem. Photobiol..

[29] Agostinis P., Berg K., Cengel K.A., Foster T.H., Girotti A.W., Gollnick S.O., Hahn S.M., Hamblin M.R., Juzeniene A., Kessel D. (2011). Photodynamic therapy of cancer: An update. CA Cancer J. Clin..

[30] Bown S.G., Rogowska A.Z., Whitelaw D.E., Lees W.R., Lovat L.B., Ripley P., Jones L., Wyld P., Gillams A., Hatfield A.W. (2002). Photodynamic therapy for cancer of the pancreas. Gut.

[31] Huggett M.T., Jermyn M., Gillams A., Illing R., Mosse S., Novelli M., Kent E., Bown S.G., Hasan T., Pogue B.W. (2014). Phase I/II study of verteporfin photodynamic therapy in locally advanced pancreatic cancer. Br. J. Cancer..

[32] Davalos R.V., Mir I.L., Rubinsky B. (2005). Tissue ablation with irreversible electroporation. Ann. Biomed. Eng..

[33] Rubinsky B., Onik G., Mikus P. (2007). Irreversible electroporation: A new ablation modality—Clinical implications. Technol. Cancer Res. Treat..

[34] Narayanan G., Bhatia S., Echenique A., Suthar R., Barbery K., Yrizarry J. (2014). Vessel patency post irreversible electroporation. Cardiovasc. Intervent. Radiol..

[35] Charpentier K.P., Wolf F., Noble L., Winn B., Resnick M., Dupuy D.E. (2010). Irreversible electroporation of the pancreas in swine: A pilot study. HPB.

[36] Zhang Z., Li W., Procissi D., Tyler P., Omary R.A., Larson A.C. (2014). Rapid dramatic alterations to the tumor microstructure in pancreatic cancer following irreversible electroporation ablation. Nanomedicine.

[37] Martin R.C.G. (2015). Irreversible electroporation of stage 3 locally advanced pancreatic cancer: Optimal technique and outcomes. J. Vis. Surg..

[38] Narayanan G., Hosein P.J., Arora G., Barbery K.J., Froud T., Livingstone A.S., Franceschi D., Rocha Lima C.M., Yrizarry J. (2012). Percutaneous irreversible electroporation for downstaging and control of unresectable pancreatic adenocarcinoma. J. Vasc. Interv. Radiol..

[39] Narayanan G., Hosein P.J., Beulaygue I.C., Froud T., Scheffer H.J., Venkat S.R., Echenique A.M., Hevert E.C., Livingstone A.S., Rocha-Lima C.M. (2017). Percutaneous Image-Guided Irreversible Electroporation for the Treatment of Unresectable, Locally Advanced Pancreatic Adenocarcinoma. J. Vasc. Interv. Radiol..

[40] Leen E., Picard J., Stebbing J., Abel M., Dhillon T., Wasan H. (2018). Percutaneous irreversible electroporation with systemic treatment for locally advanced pancreatic adenocarcinoma. J. Gastrointest. Oncol..

[41] Sugimoto K., Moriyasu F., Kobayashi Y., Saito K., Takeuchi H., Ogawa S., Ando M., Sano T., Mori T., Furuichi Y. (2015). Irreversible electroporation for nonthermal tumor ablation in patients with hepatocellular carcinoma: Initial clinical experience in Japan. Jpn. J. Radiol..

[42] Scheffer H.J., Vroomen L.G., de Jong M.C., Melenhorst M.C., Zonderhuis B.M., Daams F., Vogel J.A., Besselink M.G., van Kuijk C., Witvliet J. (2017). Ablation of Locally Advanced Pancreatic Cancer with Percutaneous Irreversible Electroporation: Results of the Phase I/II PANFIRE Study. Radiology.

[43] Ruarus A.H., Vroomen L.G.P.H., Geboers B., van Veldhuisen E., Puijk R.S., Nieuwenhuizen S., Besselink M.G., Zonderhuis B.M., Kazemier G., de Gruijl T.D. (2020). Percutaneous Irreversible Electroporation in Locally Advanced and Recurrent Pancreatic Cancer (PANFIRE-2): A Multicenter, Prospective, Single-Arm, Phase II Study. Radiology.

[44] He C., Wang J., Sun S., Zhang Y., Lin X., Lao X., Cui B., Li S. (2019). Irreversible electroporation versus radiotherapy after induction chemotherapy on survival in patients with locally advanced pancreatic cancer: A propensity score analysis. BMC Cancer.

[45] Liu S., Qin Z., Xu J., Zeng J., Chen J., Niu L., Liu S., Qin Z., Xu J., Zeng J. (2019). Irreversible electroporation combined with chemotherapy for unresectable pancreatic carcinoma: A prospective cohort study. OncoTargets Ther..

[46] Katz M.H., Fleming J.B., Bhosale P., Varadhachary G., Lee J.E., Wolff R., Wang H., Abbruzzese J., Pisters P.W., Vauthey J.N. (2012). Response of borderline resectable pancreatic cancer to neoadjuvant therapy is not reflected by radiographic indicators. Cancer.

[47] Vroomen L.G.P.H., Scheffer H.J., Melenhorst M.C.A.M., de Jong M.C., van den Bergh J.E., van Kuijk C., van Delft F., Kazemier G., Meijerink M.R. (2017). MR and CT imaging characteristics and ablation zone volumetry of locally advanced pancreatic cancer treated with irreversible electroporation. Eur. Radiol..

[48] Zhao J., Wen X., Tian L., Li T., Xu C., Wen X., Melancon M.P., Gupta S., Shen B., Peng W. (2019). Irreversible electroporation reverses resistance to immune checkpoint blockade in pancreatic cancer. Nat. Commun..

[49] White S.B., Zhang Z., Chen J., Gogineni V.R., Larson A.C. (2018). Early Immunologic Response of Irreversible Electroporation versus Cryoablation in a Rodent Model of Pancreatic Cancer. J. Vasc. Interv. Radiol..

[50] O’Brien T.J., Passeri M., Lorenzo M.F., Sulzer J.K., Lyman W.B., Swet J.H., Vrochides D., Baker E.H., Iannitti D.A., Davalos R.V. (2019). Experimental High-Frequency Irreversible Electroporation Using a Single-Needle Delivery Approach for Nonthermal Pancreatic Ablation In Vivo. J. Vasc. Interv. Radiol..

[51] Partridge B.R., O’Brien T.J., Lorenzo M.F., Coutermarsh-Ott S.L., Barry S.L., Stadler K., Muro N., Meyerhoeffer M., Allen I.C., Davalos R.V. (2020). High-Frequency Irreversible Electroporation for Treatment of Primary Liver Cancer: A Proof-of-Principle Study in Canine Hepatocellular Carcinoma. J. Vasc. Interv. Radiol..

